# Screen exposure time of children under 6 years old: a French cross-sectional survey in general practices in the Auvergne-Rhône-Alpes region

**DOI:** 10.1186/s12875-023-02009-5

**Published:** 2023-03-01

**Authors:** Mehtap Akbayin, Aurélien Mulliez, Frédéric Fortin, Mathilde Vicard Olagne, Catherine Laporte, Philippe Vorilhon

**Affiliations:** 1 Département de Médecine Générale, UFR Médecine et des professions paramédicales, Université Clermont Auvergne, 28 place Henri Dunant, 63000 Clermont-Ferrand, France; 2 Direction de la Recherche Clinique et de l’Innovation, Centre Hospitalier Universitaire de Clermont-Ferrand, F-63000 Clermont-Ferrand, France; 3 Université Clermont Auvergne, Institut Pascal, Clermont Ferrand, France; 4 Université Clermont Auvergne, UR ACCePPT, Clermont Ferrand, France

**Keywords:** Television, Digital media, Screen time, Children, General practice, Cross-sectional survey

## Abstract

**Background:**

The advent of miniature, easy-to-use and accessible multimedia products is leading to screen exposure that begins in early childhood. Overexposure in preschool may lead to adverse effects. The main objective of this study was to determine the average daily time (ADT) spent by children under 6 years of age, followed in general practice, in front of television or interactive screens.

**Methods:**

A cross-sectional survey was conducted in the Auvergne-Rhône-Alpes region among randomly selected General Practitioners (GPs). The average daily screen time (ADST), regardless of the type of device (TVs, computers, tablets, smartphones, video game consoles), of the included children aged 0 to 2 years and 2 to 5 years was calculated from a self-questionnaire completed by the parents. A multivariate Poisson regression model was performed to analyse daily screen time, adjusted by factors selected on their clinical relevance and statistical significance.

**Results:**

The 26 participating GPs included 486 parents. They reported an ADST of 26 (± 44) minutes on weekdays and 30 (± 46) minutes on weekends for children under 2 years of age. For children over 2 years of age, the ADST was 66 (± 82) minutes on weekdays and 103 (±91) minutes on weekends. There was an association between the children’s average screen time and certain sociodemographic and environmental factors. Children whose parents had higher levels of education, those living in a family without TV screens or those who were well informed about the possible adverse health consequences of overuse of screens had lower average screen time. On the other hand, children of parents who spent more than 2 hours a day in front of screens, were more exposed.

**Conclusions:**

In our survey, the ADST of children under 6 years of age followed in general practice was higher than the current recommendations. GPs can warn parents of preschool children of the effects of overexposure to screens, particularly parents of at-risk children.

## Background

Screens (televisions, computers, smartphones, tablets, video game consoles) are becoming an increasingly important part of children’s lives, starting at an early age. In the United States, before the COVID-19 pandemic, children under 2 years of age spent an average of 49 minutes per day in front of a screen and the average was 2 hours 30 minutes for children between 2 and 4 years of age [1]. Television and online videos were viewed the most by children under 6 years old, with 70% of parents believing that they were beneficial to the children [1]. In France, studies have also confirmed this exposure from an early age [2, 3].

The effects of overexposure to screens during this critical period of brain development are beginning to be understood [4]. Numerous studies have demonstrated the harmful effects of screens on the cognitive development of children [5–9] and on their academic success [10, 11]. An association between time spent in front of mobile screens and behavioural difficulties (attention disorders, hyperactivity) has been shown in preschool children [12]. It has also been shown that exposure to screens favours sedentary behaviour and therefore obesity [7, 9, 13–16]. It also impacts sleep quality [7, 9, 17–19], language development [5–7, 9] and vision [20, 21].

Based on these findings, professional recommendations advise against exposure before the age of 18–24 months and limiting it to less than 1 h per day between the ages of 2 and 5 years, giving preference to parental guidance associated with high quality and interactive programs [9, 22–24]. Primary care practitioners, paediatricians and general practitioners (GPs) can relay this advice. Few studies have been conducted on the subject in general practice. However, assessing family habits can enable GPs to provide prevention messages to parents [25]. Parental education is a modifiable factor, and interventions for parents to promote early childhood development have been shown to be effective [26] .

The primary objective of this work was to determine the daily time spent by a child under 6 years of age in front of a television or interactive screens. The secondary objectives were to assess screen use patterns in this age group and to identify factors associated with increased screen time.

## Methods

### Design

We conducted a descriptive cross-sectional survey of parents of children under 6 years of age in the Auvergne-Rhône-Alpes region in January 2019. This region, the second largest in France in terms of population size, is quite representative of the whole France considering that it contains urban areas with high population density and rural areas.

### Study population

General practices were drawn at random from the list of GPs of the Auvergne-Rhône-Alpes Regional Health Agency, and parents of children who received medical care from the selected GPs were requested to participate to the study. Random selection was made using Stata software. The objectives and modalities of the study were presented to the GPs by telephone call before obtaining their oral agreement to participate. No response after three telephone calls was considered a refusal.

Thirty questionnaires per GP were distributed. They were offered to parents by the medical secretaries or left directly in the waiting room. A collection box was made available for the participants to deposit the completed questionnaires. An information note was given to each parent, explaining the objectives of the study, the conditions of anonymization and the confidentiality of the data collected. The GPs participating in the study were contacted twice during the 4 weeks of inclusion.

All parents with at least one child under 6 years of age who agreed to participate in the survey were eligible for inclusion. Parents with several children under the age of 6 were asked to complete the questionnaire for the youngest child. Exclusion criteria were parents of children older than 6 years or those with difficulties with the French language.

### Ethics consideration

Ethical approval for the survey was obtained on the 5th of June 2019 from the Ethics Committee of the ‘Collège National des Généralistes Enseignants’ (CNGE) under number 16051998. The study was performed according to good research practices and the Declaration of Helsinki. Participants received an explanatory letter and an anonymous questionnaire by post. Written Informed consent was obtained from the participants.

### Questionnaire

We designed a questionnaire with 32 questions divided into 3 parts. It was based on the latest position statements of the French Paediatric Society (SFP) [24]. To evaluate its comprehension and acceptability, the questionnaire was pretested with 30 parents with different sociodemographic characteristics. It was composed of the following 3 parts:

- The first part included sociodemographic data about the parents: age, sex, family situation, level of education, socio-professional category, and place of residence. Two questions asked the parents about their child’s daily screen time during the week and on the weekends.
- The second part was concerned with screen equipment in the child’s home, including in their bedroom. TV screens were distinguished from other multimedia screens (smartphones, computers, video game consoles, tablets).
- The third part included questions about the child: month and year of birth, sex, the presence of older siblings, and the number of children in the household. Two questions were designed to estimate the child’s average daily screen time (ADST), both during the week and on the weekends. The parents were also asked to identify the total amount of screen time on the day before the survey to obtain greater precision and to check the consistency of the responses. Data on the children’s exposure times and parental guidance were collected. A 6-point scale was used for the responses (never, rarely, sometimes, regularly, often, always). Each of the quantifying adverbs was associated with a temporality for a better objectivity of the answers (never, 1 to 2 times per month, 1 to 2 times per week, 3 to 4 times per week, 5 to 6 times per week, every day). Finally, information that assessed the parents’ knowledge of the effects of screen misuse, the establishment of family rules and their interest in discussing the subject with a health professional was collected.

### Endpoint

The primary endpoint was the child’s ADST regardless of the type of device (TVs, computers, tablets, smartphones, video game consoles), reported by the parents in minutes and distinguishing weekdays from weekends.

### Sample size

The objective was to include 500 questionnaires. This sample size was considered to be large enough to have representative data and accurate estimations. We assumed that for a 4-week inclusion period, approximately 20 to 30 questionnaires would be collected per GP. Thus, we needed the participation of at least 25 GPs to reach 500 questionnaires. Of the 6201 general practitioners in the Auvergne-Rhône-Alpes region, 150 were selected after randomisation. In case of refusal of a general practitioner drawn at random, this one would be replaced by a doctor of the same gender (the next one in the list) in order to keep a gender balance.

### Statistical analysis

The study sample is described by numbers and percentages for categorical data and by means ± standard deviations and medians and interquartile ranges for continuous data. Daily screen time was graphically analysed, and normality was assessed using Shapiro–Wilk’s test.

Daily screen time (weekdays and weekend days) was analysed using the Mann–Whitney test for two group comparisons and the Kruskal–Wallis test for 3 (or more) groups. Relationships between daily screen time and continuous data were analysed using Spearman’s correlation coefficient.

In order to perform a multivariate Poisson regression of daily screen time, we first transformed screen time into event of exposure, considering 1 h of screen exposure as one event. Thus, children with no screen exposure were considered having 0 event, children with exposure between 1 minute and 60 minutes were considered having 1 event, children with exposure between 61 minutes and 120 minutes were considered having 2 events and so on. Then, a multivariate Poisson regression model was performed considering those screen time events as dependant variable and adjusted for factors selected according to their clinical relevance or statistical significance (*p* < 0.15) in univariate analysis. Results are shown as incidence rate ratios and their 95% confidence interval.

Analyses were performed using Stata (version 15, StataCorp, College Station).

## Results

### Description of the study population

**Fig. 1:**
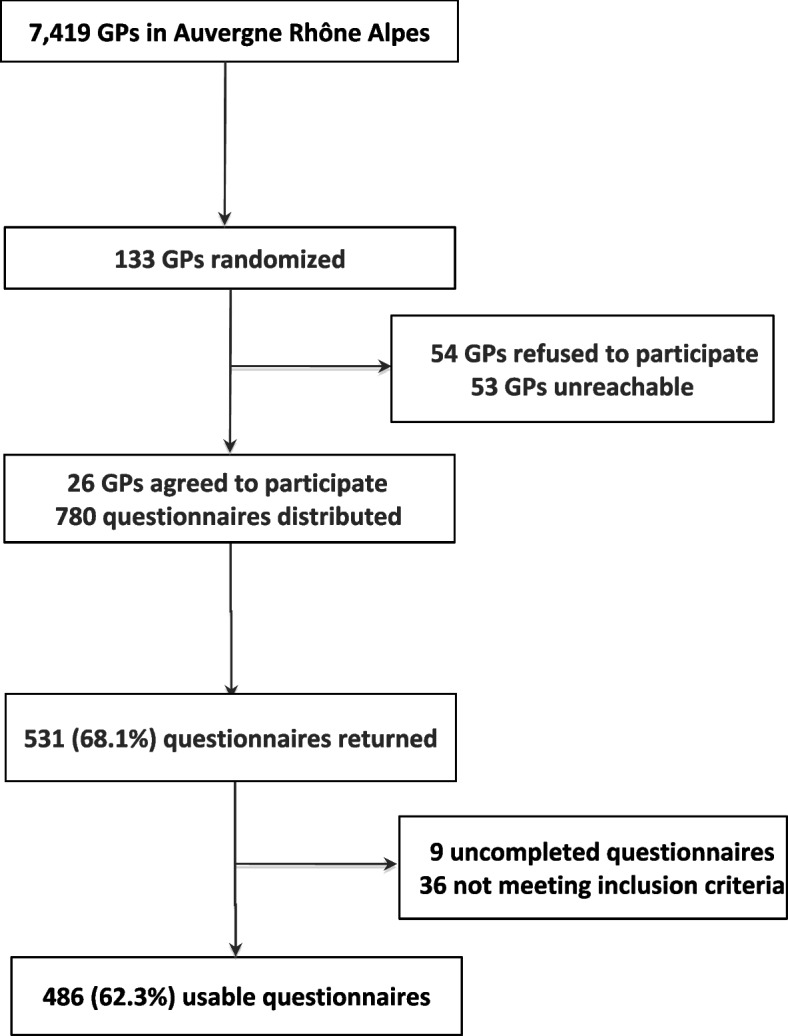
Flow-chart of the participants

**Fig. 2:**
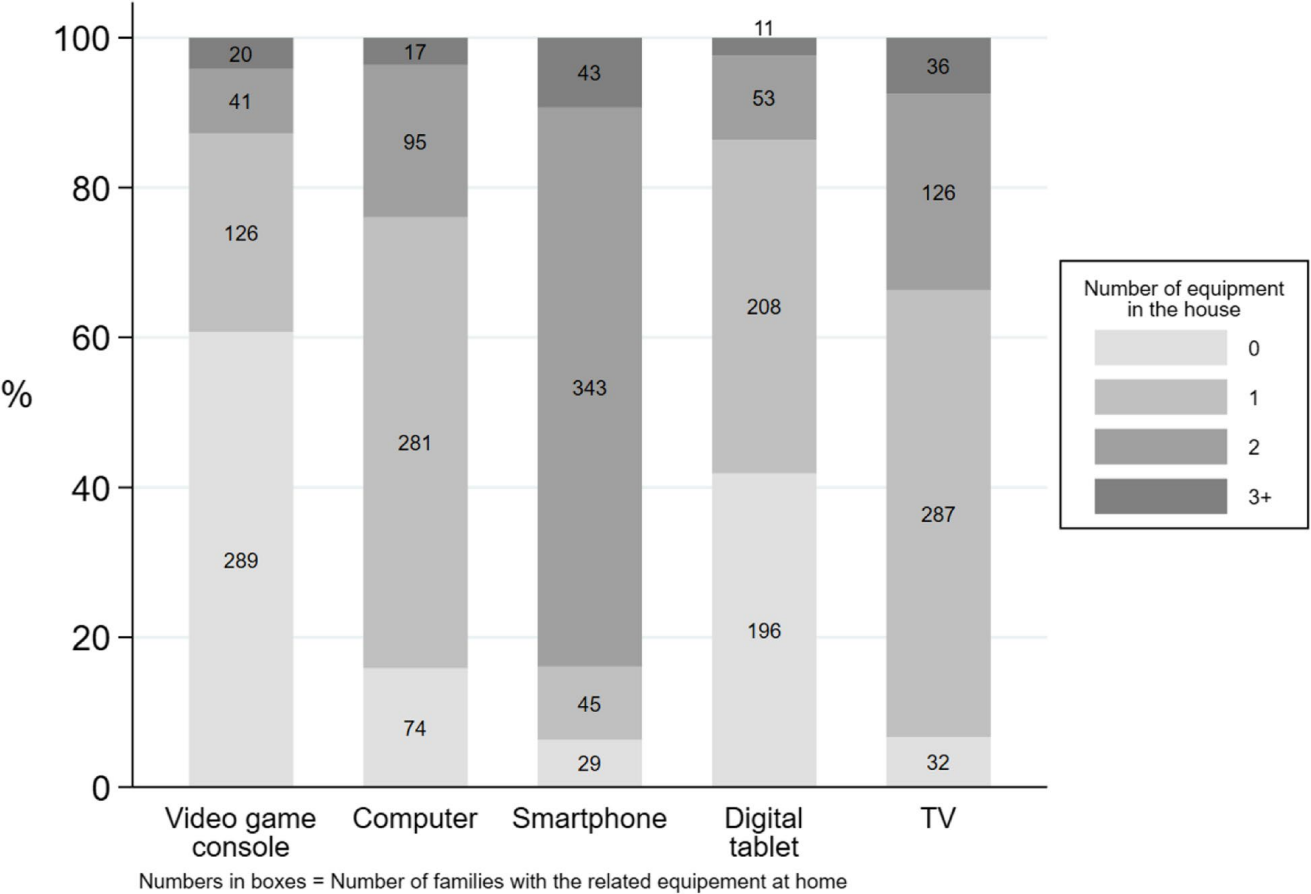
Distribution of households by type and frequency of existing screen equipment

A total of 26 GPs agreed to participate in the study. Their characteristics are described in Table 1. Among the 780 questionnaires distributed, 531 were collected, and 486 were analysed. Thirty-six patients who did not meet the inclusion criteria and 9 who partially completed the study were excluded (Fig. 1). A total of 81.2% (*n* = 394) of the questionnaires were completed by mothers; the mean age of the respondents was 34 years (± 5.1). Among the children, 46.1% (*n* = 224) were girls; the mean age was 3.7 years (±1.5) (Table 2). A total of 449 households (93.4%) reported owning at least one television set (Fig. 2). In addition, 431 families (93.7%) reported owning at least one smartphone, 393 reported owning at least one computer (84.2%) and 208 reported owning a tablet (44.4%).

**Table 1:** Characteristics of participating general practitioners

Variables	Sample *n* = 26	Overall (2018) *n* = 7419	*P*-value
Sex, n (%)			
Female	13 (50)	3340 (45.0)	0.61
Male	13 (50)	4079 (55.0)	
Age, mean (sd)	45.6 ± 13	52.0 (± NA^*^)	NC^*^
Number of years of practice	14.2 ± 14.0	17.5 (± NA^*^)	NC^*^
Location, n (%), (*n* = 7174)			
Rural	6 (23.1)	1038 (14.5)	0.21
Urban	20 (76.9)	6136 (85.5)	
N missing	0	245	
Type of practice			
Individual practice, n (%)	12 (46.1)	4664 (62.9)	0.08
Group practice, n (%)	14 (53.8)	2755 (37.1)	
Other			
GP teacher, n (%)	7 (26.9)	1590 (21.4)	0.50

Health insurance data, as of 31 January 2018

^*^NA Not available, *NC* Not computable

**Table 2:** Characteristics of the study population and daily weekdays screen time

Sample characteristics	*N* = 486	Weekdays screen time (minutes) Median [IQR]*	*P* value
Children, *n* = 486			
Age, mean ± standard deviation (sd)	3.7 ± 1.5		
≤ 2 years, n (%)	75 (15.4)	0 [0–30]	< 0.001
2–6 years, n (%)	411 (84.6)	50 [20–90]	
Gender			
Female, n (%)	224 (46.1)	30 [5–60]	0.02
Male, n (%)	262 (53.9)	48 [20–90]	
Older siblings			
Yes n (%)	262 (54.1)	43 [15–90]	0.18
No, n (%)	222 (45.9)	30 [10–60]	
TV in bedroom			
Yes n (%)	27 (5.6)	120 [60–150]	< 0.001
No, n (%)	456 (94.4)	30 [10–60]	
Parents, *n* = 486			
Age, mean ± sd	33.9 ± 5.1		
Number of children, mean ± sd	2.01 ± 0.81		
1, n (%)	125 (25.7)	30 [5–60]	0.077
2, n (%)	261 (53.7)	30 [15–90]	
3, n (%)	77 (15.5)	60 [30–60]	
≥ 4, n (%)	23 (4.7)	60 [20–120]	
Gender			
Female, n (%)	394 (81.2)	30 [12–60]	0.83
Male, n (%)	91 (18.8)	45 [15–66]	
*Family situation*			
In couple, n (%)	438 (90.3)	30 [10–60]	< 0.001
Alone, n (%)	47 (9.7)	60 [30–120]	
*Residence*			
*Urban,* n (%)	265 (54.5)	30 [10–90]	0.81
Rural, n (%)	221 (45.5)	30 [20–60]	
*Socio-professional category*			
Farmers, n (%)	2 (0.4)	40 [20–60]	< 0.001
Craftsmen, merchants, business managers, n (%)	25 (5.2)	20 [0–60]	
Executives and intellectual professions, n (%)	104 (21.6)	30 [2–60]	
Intermediate professions, n (%)	39 (8.1)	30 [5–60]	
Employees, n (%)	205 (42.5)	50 [20–90]	
Workers, n (%)	23 (4.8)	60 [30–120]	
Retired, n (%)	2 (0.4)	165 [150–180]	
No activty, n (%)	82 (17)	60 [30–120]	
*Education level*			
Primary School	24 (5.0)	60 [45–120]	< 0.001
Secondary School	158 (32.8)	60 [30–120]	
Bachelor’s degree or above	299 (62.2	30 [5–60]	

^*^Medians expressed in minutes

### Average Daily Screen Time (ADST)

**Fig. 3:**
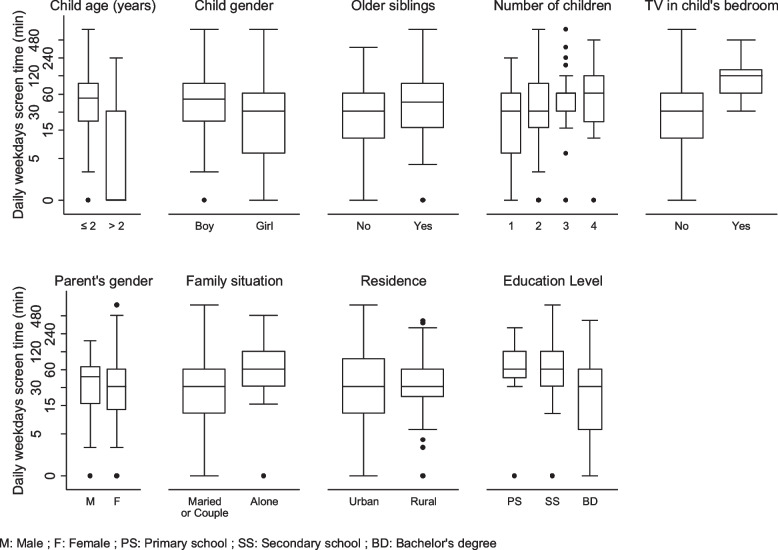
Children daily weekdays screen time according to their characteristics

The ADST for children under 2 years of age on weekdays was 26 minutes (±44.0), with a median IQR of 0 [0–30] (see Table 2 and Fig. 3) and that on weekends was 30 minutes (±46.9), with a median IQR of 0 [0–50]. The ADST of children older than 2 years on weekdays was 66 minutes (±82.1), with a median IQR of 50 [20–90] and that on weekends was 103 minutes (±91.3), with a median IQR of 90 [45–120]. Both daily weekday and weekend average screen times reported by the parents were significantly higher for boys than for girls (weekday with a median IQR of 48 [20–90], weekend days with a median IQR of 30 [5–60], *P* = 0.02, respectively) (Fig. 3). The ADST increased significantly with the age of the child (*p* < 0.001).

According to the parents, 316 children (66%) had watched a TV and/or multimedia screen the day before the questionnaire. The previous day’s average screen time was 43 minutes (±57) across all age groups. Children’s ADSTs increased concomitantly with parents’ ADSTs, both on weekdays (correlation coefficient *r* = 0.31, *p* < 0.001) and weekends (*r* = 0.37, *p* < 0.001).

### Children’s screen use habits

According to the parents, 456 children (94.4%) did not have a screen in their room, and 443 children (91.5%) never used a multimedia screen in their room. Children who had a TV in their room had higher weekday and weekend ADSTs than those who did not (124 ± 91.8, median 120 [60–150] vs. 56.0 ± 76.6, median 30 [10–60], *P* < 0.001 on weekdays and 187 ± 131, median 150 [120–240] vs. 86.0 ± 83.7, median 60 [30–120], *P* < 0.001 on weekends, respectively) (see Table 2 and Fig. 3). One hundred and two parents (25.4%) said they never discussed the content of the program their child watched, and for 72 (14.8%) of them, the child was regularly or always alone in front of a screen.

Concerning TV, 119 parents (24.7%) estimated that their child watched TV during the week before school, and 62 parents (12.7%) watched TV in the evening before bedtime. Furthermore, 53 children (10.9%) were regularly present when their parents watched TV, and 45 children (9.2%) were always present. In 63 households (12.9%), the television was always on during meals. Finally, 78.8% of the parents in our study wanted to discuss screen recommendations with their general practitioner.

### Factors associated with increased screen time – multivariate analysis

**Fig. 4:**
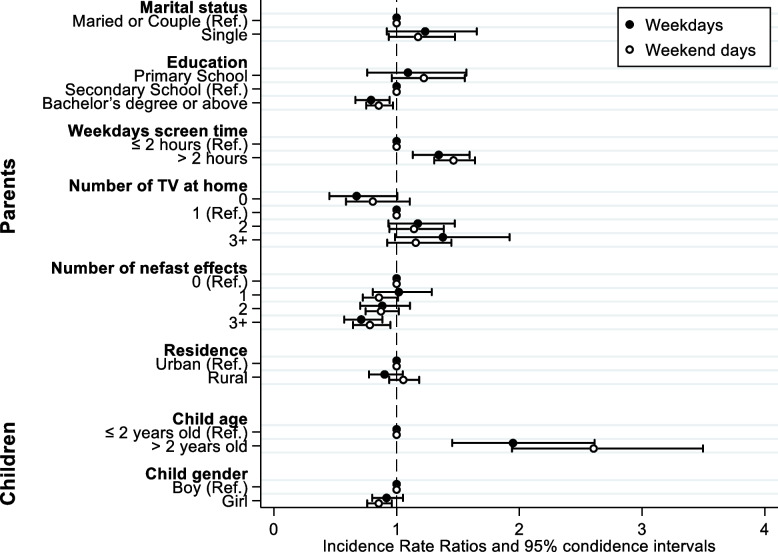
Factors associated with weekdays and weekend days screen time, according to multivariate Poisson regression

The multivariate Poisson regression model adjusted for clinically relevant criteria showed that, compared to Secondary School parents, the higher the parents’ level of education, the less the children watched a screen (IRR = 0.79, 95%CI [0.66–0.94] (Tables 3 and 4 and Fig. 4). Compared to one TV at home (reference), on weekdays, children with no TV were less exposed (IRR = 0.67, 95%CI [0.45–1.01], *p* = 0.053), than children with three or more television screen (IRR = 1.38, 95%CI [0.99–1.92], *p* = 0.059). As well on weekdays as on weekends, when parent’s daily screen time was > 2 hours, children exposure increased, with IRR = 1.34, 95%CI [1.13–1.59], *p* = 0.001. Compared to parents who were not aware of any harmful effects of screen overexposure considered as reference, the children of parents who were aware of at least 3 adverse effects had lower ADSTs during the weekdays and weekends. Finally, children from single-parent families tend to be more exposed than others on weekdays with IRR = 1,23, 95% CI [0.9–1.65], *p* = 0.162 and weekends with IRR = 1.17, 95% CI [0.9–1.47], *p* = 0.165 and weekends.

**Table 3:** Multivariate Poisson regression of weekdays exposure

	*IRR*	95% CI	*P* value
*Family situation*			
Maried or Couple (Ref.)			
Single	1.23	[0.9–1.65]	0.162
*Education level*			
Primary School	1.09	[0.8–1.57]	0.633
Secondary School (Ref.)			
Bachelor’s degree or above	0.79	[0.7–0.94]	0.009
*TEM parents*			
Parent’s screen time ≤ 2 hours (Ref.)			
Parent’s screen time > 2 hours	1.34	[1.1–1.59]	0.001
*Number of TV at home*			
0	0.67	[0.5–1.01]	0.053
1 (Ref.)			
2	1.17	[0.9–1.47]	0.173
3+	1.38	[1–1.92]	0.059
*Number of nefast effects*			
0 (Ref.)			
1	1.02	[0.8–1.29]	0.881
2	0.88	[0.7–1.11]	0.286
3+	0.71	[0.6–0.88]	0.002
*Residence*			
Urban (Ref.)			
Rural	0.9	[0.8–1.05]	0.185
*Child age*			
≤ 2 years old (Ref.)			
> 2 years old	1.95	[1.5–2.61]	< 0.001
*Child gender*			
Boy (Ref.)			
Girl	0.92	[0.8–1.05]	0.218

**Table 4:** Multivariate Poisson regression of weekend exposure

	*IRR*	95% CI	*P* value
*Family situation*			
Maried or Couple (Ref.)			
Single	1.17	[0.9–1.47]	0.165
*Education level*			
Primary School	1.22	[1–1.56]	0.103
Secondary School (Ref.)			
Bachelor’s degree or above	0.85	[0.8–0.97]	0.016
*TEM parents*			
Parent’s screen time ≤ 2 hours (Ref.)			
Parent’s screen time > 2 hours	1.46	[1.3–1.64]	0
*Number of TV at home*			
0	0.81	[0.6–1.11]	0.184
1 (Ref.)			
2	1.14	[0.9–1.38]	0.177
3+	1.16	[0.9–1.45]	0.205
*Number of nefast effects*			
0 (Ref.)			
1	0.86	[0.7–1.01]	0.068
2	0.87	[0.7–1.02]	0.084
3+	0.78	[0.6–0.95]	0.013
*Residence*			
Urban (Ref.)			
Rural	1.05	[0.9–1.18]	0.365
*Child age*			
≤ 2 years old (Ref.)			
> 2 years old	2.6	[1.9–3.5]	< 0.001
Child gender			
Boy (Ref.)			
Girl	0.85	[0.8–0.96]	0.009

## Discussion

### Main results

Our study which focuses on parents of children followed in general practice, highlights that children of parents with Bachelor’s degree or above education level were less exposed to screens. On weekdays, those without TV at home had less screen time. On the other hand, the children of parents who were well informed about the possible adverse health consequences of overuse of screens had lower average screen time. On weekend days, the ADST of girls was less important than boys. The ADST tended to increase among children from single-parent families on weekdays and weekend days. Finally, there was no difference by rural or urban location.

### Comparison with literature data

We did not find similar studies among a population of patients followed in general practice. Several studies in primary care involved patients followed in paediatric clinics or offices [2, 27]. In our study, both weekday and weekend ADSTs were lower than those reported in American data from 2020 [1]. Indeed, before the COVID-19 pandemic and the implementation of the first containment, American children under 2 years of age spent an average of 49 minutes per day in front of screens. Those aged 2 to 4 years had a daily screen time of 150 minutes per day. It should be explain by the fact that our data predate the American data and that all studies tend to show a steady increase in screen time among children [12, 14, 28, 29]. Our results for children under 2 years of age are also lower than those of an Australian study published in 2016 that revealed a daily screen time of more than 2 hours for 40% of 18-month-olds [30]. This difference might be caused by our age range including children younger than 18 months with less screen time.

Our study took place just before the Covid 19 pandemic. It can be assumed that the Covid pandemic changed children’s screen use behaviours, at least for older children. During the first wave of the pandemic, some studies assessed screen time among preschoolers. In an international study, parents reported an average increase of nearly 1 h of screen time per day in 3- to 7-year-old children [31]. This increase was largely due to their use for entertainment purposes. Another study, by Fitzpatrick et al., found an increase in screen time specially before bedtime [32]. In this study, children’s age and parental use of multimedia screens were factors associated with increased screen time, but teleworking parents were less likely to have overexposed children. Not surprisingly, older children with online schooling requirements spent more time in front of a screen at first containment. In an other international survey in children under the age of three, this increase in screen time was also confirmed during the first lockdown [33].

Our study also found that some environmental factors may influence children’s screen time.

In similar studies, the number of TV screens in the home is associated with children’s screen time [34, 35]. In the same way, living in a single-parent family is a contributing factor to increased screen time. However, although there is an upward trend in our study, the association is not significant. One of the explanations for this is that screens can be a means of distracting or calming children when the parent is engaged in certain tasks, which would seem to be even more valid in a single-parent family [28, 36].

In studies, children’s screen time is commonly associated with parental screen time [29, 35]. Lauricella et al. found that the amount of time parents used a multimedia technology (computers, tablets, smartphones) was associated with the amount of time the child used that same technology [37]. In other words, parental behaviours and habits with respect to multimedia screens strongly influence those of the children. Birkin et al. and Matta et al. have established that the regulation of their own use of new technologies by parents allows to avoid the reproduction of harmful behaviours by their children. This can be done by setting up parental rules [35, 38].

Parental education level is associated with children’s screen time [14, 38]. Atkin et al. found that children of mothers with low levels of education were more likely to exceed 2 h/day of screen time [14] and for Kiliç et al. the frequency of tablet use and ownership among children was inversely related to maternal education and household income [28]. But for Paudel et al. review, the association between educational status and children’s mobile screen media use is not really demonstrated [39].

Some studies showed that children’s mobile screen use increases when parents perceive beneficial effects and educational value [37]. Similarly, negative parental beliefs about screen-based mobile media are associated with decreased screen use [40]. In addition, a French study conducted in 2019 showed that parents were mainly misinformed about the risk of obesity [25]. Finally, our data demonstrate that the more knowledge parents had about the harmful effects associated with overuse of screens, the more children’s screen time decreased. This finding is particularly interesting because there are relatively few data in the literature on this aspect.

### Strengths and limitations of the study

The number of parents included reached 500, giving enough statistical power to the study. The final sample of GPs was representative of the region’s GPs for sex, age, and practice location. However, the results must be interpreted considering a number of limitations.

The survey was systematically proposed to parents of children under 6 years of age, but this did not exclude a possible selection bias by the secretaries or GPs offering the questionnaires. Furthermore, parents who were involved and aware of this subject could easily agree to answer the questionnaire. Another selection bias was related to the nonparticipation of populations with a language barrier. The effect of non-participating population is difficult to interpret regarding the data available in the literature. This population may include people with low education level, and/or migrants. We could make the assumption that this exclusion underestimates the screen time of our population. Indeed, in her study about children under 3 years of age, Duch showed a positive correlation between screen time and ethnic minority status [41]. This, together with the low rate of participating GPs, limits the extrapolation of the results to the entire French paediatric population.

The questionnaires were anonymously completed by the patients before or after the consultation and placed in a collection box. This method allowed the parents to be as honest as possible in their answers, but as this was a declarative survey, it does not exclude a possible social desirability bias, which could lead to a minimisation of the ADST.

For our primary endpoint, we used the parents’ global memory to determine children’s ADSTs on weekdays and weekends. A memory bias is possible because of the difficulty of providing synthetic and global data for a usual practice. This bias can be balanced by the fact that the parents’ assessments of the previous day’s screen time across all age groups was lower than their overall estimates of daily weekday and weekend average screen times. In addition, our study did not assess daily exposure times for every type of screen.

### Perspectives on care

An Australian study found that health-related habits in families crystallise most easily in early childhood [42]. Thus, educational measures regarding sensible screen use should be implemented in early childhood to promote appropriate use. In our study, 45% of the parents had established rules for the use of screens, and the children of parents who were aware of several harmful effects had less screen time. If we add that nearly 80% of the parents would like more information on the subject, these data should encourage primary care practitioners, especially GPs, to screen children at risk of overexposure and to sensitize their families to implement preventive measures.

## Conclusion

Our study of parents of preschool children followed in general practice showed early exposure to screens with daily average screen times beyond the professional recommendations. Intervention by GPs is legitimate to assess screen use and identify children at risk of overuse. Communication with families can provide the explanations that parents often expect and develop prevention advice.

## Data Availability

The datasets used and/or analysed during the current study are available from the corresponding author up on reasonable request.

## Abbreviations

GPs: General Practitioners
ADT: Average daily time
ADST: Average daily screen time
FPS: French Pediatric Society
